# Ultra-early tranexamic acid after subarachnoid hemorrhage (ULTRA): study protocol for a randomized controlled trial

**DOI:** 10.1186/1745-6215-14-143

**Published:** 2013-05-16

**Authors:** Menno R Germans, René Post, Bert A Coert, Gabriël JE Rinkel, W Peter Vandertop, Dagmar Verbaan

**Affiliations:** 1 Department of Neurosurgery, Neurosurgical Center Amsterdam, Academic Medical Center, PO Box 22660, Amsterdam, 1100 DD, the Netherlands; 2 Department of Neurology and Neurosurgery, Rudolf Magnus Institute of Neuroscience, University Medical Center Utrecht, PO Box 85060, Utrecht, 3508 AB, the Netherlands

**Keywords:** Neurosurgery, Subarachnoid hemorrhage, Early medical intervention, Tranexamic acid, Antifibrinolytic agents, Intracranial aneurysm, Functional outcome, Rebleeding

## Abstract

**Background:**

A frequent complication in patients with subarachnoid hemorrhage (SAH) is recurrent bleeding from the aneurysm. The risk is highest within the first 6 hours after the initial hemorrhage. Securing the aneurysm within this timeframe is difficult owing to logistical delays. The rate of recurrent bleeding can also be reduced by ultra-early administration of antifibrinolytics, which probably improves functional outcome. The aim of this study is to investigate whether ultra-early and short-term administration of the antifibrinolytic agent tranexamic acid (TXA), as add-on to standard SAH management, leads to better functional outcome.

**Methods/Design:**

This is a multicenter, prospective, randomized, open-label trial with blinded endpoint (PROBE) assessment. Adult patients with the diagnosis of non-traumatic SAH, as proven by computed tomography (CT) within 24 hours after the onset of headache, will be randomly assigned to the treatment group or the control group. Patients in the treatment group will receive standard treatment with the addition of a bolus of TXA (1 g intravenously) immediately after randomization, followed by continuous infusion of 1 g per 8 hours until the start of aneurysm treatment, or a maximum of 24 hours after the start of medication. Patients in the control group will receive standard treatment without TXA. The primary outcome measure is favorable functional outcome, defined as a score of 0 to 3 on the modified Rankin Scale (mRS), at 6 months after SAH. Primary outcome will be determined by a trial nurse blinded for treatment allocation. We aim to include 950 patients in 3 years.

**Discussion:**

The strengths of this study are: 1. the ultra-early and short-term administration of TXA, resulting in a lower dose as compared to previous studies, which should reduce the risk for delayed cerebral ischemia (DCI), an important risk factor in the long-term treatment with antifibrinolytics; 2. the power calculation is based on functional outcome and calculated with use of recent study results of our own population, supported by data from prominent studies; and 3. the participation of several specialized SAH centers, and their referring hospitals, in the Netherlands with comparative treatment protocols.

**Trial registration:**

Nederlands Trial Register (Dutch Trial Registry) number NTR3272

## Background

Subarachnoid hemorrhage (SAH) from a ruptured aneurysm occurs in relatively young patients (mean age 55 years) and accounts for 5% of all strokes, with an incidence of approximately 9 per 100,000 person-years [1,2]. The case fatality rate is approximately 35%, 25% of the survivors have a favorable outcome, and only a small group recover completely [3]. A frequent complication in patients with a SAH is recurrent bleeding from the aneurysm, which happens primarily within the first few hours after the initial hemorrhage, and occurs in 10% to 22% of patients who present to a hospital [4-9]. Besides the primary hemorrhage, recurrent bleeding is still one of the major causes of death and disability [10].

Rebleeding can be prevented by early aneurysm occlusion, but in daily clinical practice treatment is often delayed by logistical factors, such as delay in diagnosis or transfer between hospitals [11-13] (own data, manuscript in preparation). Therefore, early aneurysm treatment alone is not sufficient to prevent all recurrent bleedings and other strategies have to be explored. An alternative to reduce the risk of rebleeding is the administration of an antifibrinolytic agent, which slows the breakdown of the blood clot, prior to aneurysm occlusion [14]. Long-term administration of antifibrinolytics has been found to effectively reduce the risk of recurrent bleeding by approximately 40%, but patient outcome does not improve owing to a concurrent increase in delayed cerebral ischemia (DCI) [14]. Recent studies using early and short-term antifibrinolytic therapy have also shown reduction of recurrent rebleeding but without an increase in DCI [7,8,14,15]. However, the only randomized controlled trial (RCT) that was performed was underpowered to show an effect on functional outcome [7].

The aim of this RCT is to investigate whether treatment with ultra-early and short-term administration of the antifibrinolytic agent tranexamic acid (TXA), as add-on to standard, state-of-the-art SAH management, leads to a significantly higher percentage of patients with a favorable functional outcome, defined as a score of 0 to 3 on the modified Rankin Scale (mRS), assessed at 6 months after SAH.

## Methods/Design

### Design

The ultra-early tranexamic acid after subarachnoid hemorrhage (ULTRA) study will be performed as a multicenter, prospective, randomized, open-label trial with blinded endpoint (PROBE) assessment.

### Setting

The protocol of this study was approved by the Medical Ethics Committee (MEC) of the Academic Medical Center (AMC), Amsterdam, the Netherlands. It is registered at the Nederlands Trial Register (Dutch Trial Registry), number NTR3272. At the start of the study at 15 May 2013, a total of 26 participating hospitals, including three specialized SAH centers (study centers) and their referring hospitals, will start randomization for the ULTRA study. It is expected that four additional study centers and their referring hospitals will start randomization in the near future.

### Participants

All patients presenting with a proven SAH will be checked for eligibility by the treating physician. Inclusion criteria are: adult patients (≥18 years) with the diagnosis of SAH, as proven by CT within 24 hours after the last hemorrhage. Exclusion criteria are: 1. no loss of consciousness after the hemorrhage, with World Federation of Neurological Surgeons (WFNS) grade 1 or 2 on admission in combination with a perimesencephalic bleeding pattern; 2. history and bleeding pattern on CT compatible with a traumatic SAH; 3. ongoing treatment for deep vein thrombosis or pulmonary embolism; 4. history of a hypercoagulability disorder; 5. pregnancy; 6. severe renal (serum creatinine >150 mmol/l) or liver (aspartate aminotransferase (AST) >150 U/l or alanine aminotransferase (ALT) >150 U/l or alkaline phosphatase (ALP) >150 U/l or gamma-glutamyltransferase (γ-GT) >150 U/l) failure; 7. expected death within 24 hours; and 8. participation in another SAH intervention study.

### Randomization

Online randomization will be performed by the treating physician in the study center using permuted blocks and with stratification by study center, to ensure an equal number of patients in both study arms at each study center.

### Interventions

**Figure 1:**
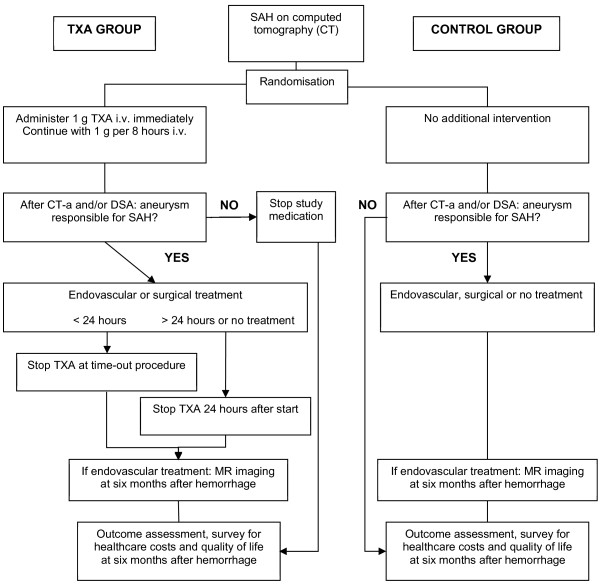
Flow chart of procedures.

All participating patients will be treated according to state-of-the-art SAH management, comparable with recent published international guidelines [2,16]. Subjects with CT-confirmed SAH who are randomly assigned to the treatment group will receive additional administration of TXA. This treatment consists of a bolus of TXA (1 g intravenously) as soon as possible after randomization (also at the referring hospital when applicable), followed by continuous infusion of 1 g per 8 hours, until a maximum of 24 hours after the start of medication. If aneurysm treatment is initiated within 24 hours after the TXA bolus, the medication infusion will be discontinued at the time-out procedure before start of the aneurysm treatment (endovascular or surgical). The continuous infusion of the study medication will be cancelled immediately if, after inclusion: 1. no aneurysm appears to be present on CT angiography (CTA) and/or digital subtraction angiography (DSA); 2. other intracranial pathology than an aneurysm is responsible for the SAH; or 3. the aneurysm which is visualized is not held responsible for the hemorrhage, based on the bleeding pattern on CT. Patients randomly assigned to the control group will not receive TXA treatment. The procedures performed during the study are outlined in Figure 1.

### Consent procedure

This study evaluates the impact of a treatment initiated as soon as possible in an emergency situation. Since the majority of patients will not be able to give informed consent at admission, the informed consent procedure for this study will be delayed in a so-called emergency procedure. For patients who are randomized for TXA administration, the medication will be administrated as soon as possible after randomization. Information about the study will be given to the patients or their legal representatives as soon as possible in the study center. Patients or their legal representatives will be asked about participation in the study by signing an informed consent. If patients or legal representatives decline to participate, study medication will be stopped immediately (if randomized for treatment and TXA is still administered) and the patient will be excluded from the study.

### Primary outcome

The primary endpoint, defined as a favorable functional outcome on the mRS (score 0 to 3) [17], will be assessed at 6 months after the SAH by a trial nurse who is blinded for treatment allocation during a standardized, validated telephone interview [18].

### Secondary outcomes

Secondary outcome measures are: case fatality rate, cause of poor outcome, rebleeding rate, complication rate (including delayed ischemic stroke, thromboembolic events, hydrocephalus, extracranial thrombosis, or hemorrhagic complications), discharge location, (micro)infarctions on magnetic resonance (MR) imaging at 6 months after endovascular treatment, quality of life at 6 months after SAH (with EQ-5D questionnaire (EuroQol Group, Rotterdam, the Netherlands) [19]), and healthcare use and healthcare-related costs until 6 months after SAH.

### Sample size calculation

The primary endpoint analysis of this study is based on the difference in percentage of patients with favorable outcome (mRS score 0 to 3) at 6 months after SAH between patients with and without TXA treatment. This expected difference between the TXA and control group was estimated with the results of renowned SAH studies and our own data (293 consecutive aneurysmal SAH patients, added with angiogram-negative SAH patients, treated at the AMC between 2008 and 2011). The percentage of SAH patients (including angiogram-negative patients) who reach the hospital have a favorable outcome of 69% (own data) and the rebleeding rate is 17%, which is consistent with numbers reported in previous studies (11% to 22%) [6-8]. For patients with recurrent bleeding, an estimated 20% will have a favorable outcome (0.17 × 0.20 = 3.4% of the total control group). Consequently, the percentage of patients with a favorable outcome without recurrent bleeding is 79% (total of patients without recurrent bleeding with a favorable outcome: 69 - 3.4 = 65.6%; total of patients without recurrent bleeding: 100 - 17 = 83%; 65.6 / 83 = 0.79).

In the TXA group, the reduction in recurrent bleeding is expected to be 77% [7,8], which reduces the rate of rebleeding to 3.9% (0.17 × 0.77 = 13.1%; 17% - 13.1% = 3.9%). Furthermore, TXA is anticipated to improve the percentage of favorable outcome in patients with recurrent bleeding from 20% to 30% [7]. Therefore, in the TXA group, 3.9% will have recurrent bleeding, of which 30% will have a favorable outcome (0.039 × 0.3 = 1.2% of the total TXA group). Patients without recurrent bleeding will have a favorable outcome of 79%, which is 75.9% of the total TXA group (total of patients without recurrent bleeding: 100 - 3.9 = 96.1; 0.961 × 0.79 = 75.9%). In the TXA group, the total of patients with a favorable outcome is 77.1% (75.9% + 1.2%). Based on these assumptions, it is expected that TXA administration will increase the proportion of patients with a favorable outcome from 69% to 77.1%. A two-group chi-square test with a 0.05 two-sided significance level will have 80% power to detect the difference between a control group proportion of 0.69 and a treatment group proportion of 0.771 (odds ratio of 1.513) when the sample size in each group is 470 (940 patients in total). The plan is to include a total of 950 patients. The aim is to include these patients within 3 years. Analysis of the results is planned in 2016.

### Statistical analysis

The statistical analysis will be by intention-to-treat. The occurrence of the primary outcome, mRS score 0 to 3 at 6 months, will be compared between the two randomization groups. The secondary outcome analyses will compare the above described variables between randomization groups. Subgroup analyses will be performed to evaluate whether the rate of recurrent bleeding and percentage of favorable outcome differ between gender and groups with different WFNS grade at admission. Subsequently, the association between favorable outcome and time interval from the last hemorrhage to first TXA administration will be evaluated. Group differences for continuous variables will be calculated using an independent t-test for continuous variables with a parametric distribution or Mann-Whitney U test for continuous variables with a non-parametric distribution. Group differences for categorical variables will be calculated using chi-square statistics. Chi-square statistics will be used to calculate an odds ratio, risk ratio or risk difference. Adjustments for factors that differ at randomization will be made using regression or multi-level models. A *P* value <0.05 will be considered significant.

For the cost-effectiveness analysis, the mRS score at 6 months is the effect measure. The costs during admission and the costs from discharge to 6 months after SAH will be summed up, resulting in one value for overall direct and indirect costs. The incremental cost-effectiveness ratio and its 95% confidence interval (CI) will be estimated with bootstrapping.

### Data safety analysis

An interim analysis will be performed by a Data and Safety Monitoring Board (DSMB) when half of the patients (n = 475) are enrolled. In this analysis unblinded data are assessed and the DSMB can advise to adjust the conduct, design or sample size, or to terminate the study according to predefined stopping rules. These rules are: 1. clear evidence that TXA administration is harmful for patients; or 2. superiority in functional outcome (mRS 0 to 3) in the treatment group after 6 months, evaluated with the Peto analysis [20] with a *P* value of 0.001.

## Discussion

Antifibrinolytic therapy after aneurysmal SAH reduces rebleeding by approximately 40% [14], but outcome is not improved owing to a concurrent increase in DCI. Recent studies of early, short-term antifibrinolytic therapy have shown better results, with a tendency for improved functional outcome, but were judged to be biased and, in case of the only RCT, underpowered [7,8,15]. These results raise the expectation that a reduction of rebleeding without an increase of DCI will improve functional outcome at 6 months. We have developed an RCT for ultra-early and short-term treatment with TXA, with the sample size calculation based on the expected improvement in functional outcome.

Our study has several strengths. TXA can be administrated as soon as possible after randomization since the emergency procedure for delayed informed consent is used in this study, and the risk of DCI is reduced to a minimum by the short-term and low total dose of TXA. In addition, our dosing regimen is considered safe because the maximum dose is lower than in previous studies and the administration regimen is in concordance with the CRASH-2 trial [21]; the power calculation is based on functional outcome and calculated with use of recent study results of our own population, supported by data from prominent studies; a significant proportion of the ten specialized SAH centers throughout the Netherlands, including their referring hospitals, are willing to participate; and both coordinating centers (AMC and University Medical Center Utrecht (UMCU), the Netherlands) are experienced in coordinating clinical trials in SAH.

There are some limitations of our protocol. First, patients and personnel are not blinded for treatment allocation, which could potentially lead to a detection bias. However, owing to the PROBE design, the assessor of the primary endpoint is blinded for treatment allocation, which prevents detection bias. Second, the maximum duration of TXA administration will be 24 hours. Theoretically, this could be too short for patients who are treated later than 24 hours after the diagnosis. However, the risk of rebleeding is strongly decreased after 24 hours [6] and the antifibrinolytic function of TXA in blood serum remains for 7 to 8 hours when several doses have been given. In addition, our own data (not shown) show that 74% of patients are treated within 24 hours, with a median time of 18 hours. Therefore, with the early aneurysm treatment, low risk of rebleeding after 24 hours and remaining antifibrinolytic function, we expect to expose patients to a minimal risk of rebleeding when terminating TXA therapy after a maximum of 24 hours.

In conclusion, we have developed a protocol for a randomized, open-label study with administration of TXA in SAH patients. In contrast with earlier studies, the treatment will start ultra-early to reduce as many recurrent bleedings as possible and treatment will be continued to a maximum of 24 hours to prevent an increase in DCI. Since functional outcome assessed at 6 months is taken as the primary endpoint, this study will provide an answer for whether an increase of favorable outcome can be reached in patients with ultra-early and short-term TXA treatment after SAH.

## Roles and responsibilities

### Principal investigator and executive team

D Verbaan is the principal investigator (PI) and will coordinate the trial. D Verbaan is responsible for preparation of protocols and questionnaires, recruiting centers and funding, MEC application, day-to-day conduct of the trial, supplying information about the trial, statistical validity, and publication of the results. MR Germans is responsible for preparation of protocols and questionnaires, recruiting funding, MEC application, evaluating cause of unfavorable outcome, data analysis, writing, and publication of the results. BA Coert is responsible for recruiting centers, medical support in day-to-day conduct of the trial, evaluating cause of unfavorable outcome, and publication of the results. R Post is responsible for recruiting centers, day-to-day conduct of the trial, trial database, communicating and writing information to centers, preparing DSMB and annual reports, data analysis, writing, and publication of the results. Prof WP Vandertop is the PI at the AMC and VU University Medical Center (VUMC), the Netherlands, and is responsible for evaluating cause of unfavorable outcome, and publication of the results. Prof GJE Rinkel is PI at the UMCU. NM Fleitour is responsible for entering data in the trial database, coordinating all correspondence, and sending postal questionnaires and reminders. YK Bartels will perform the telephone interview for outcome assessment at 6 months.

## Trial committees

### Executive committee

D Verbaan, PhD (PI, AMC); MR Germans, MD (study coordinator (SC), AMC); BA Coert, MD, PhD (SC, AMC); R Post, MD (SC, AMC); Prof WP Vandertop, MD (PI, AMC, VUMC); Prof GJE Rinkel, MD (PI, UMCU); NM Fleitour (trial nurse); and YK Bartels (outcome assessor).

### Steering committee

D Verbaan, clinical epidemiologist (AMC); MR Germans, neurosurgeon (AMC); BA Coert, neurosurgeon (AMC); R Post, resident neurosurgery (AMC); Prof WP Vandertop, neurosurgeon (AMC, VUMC); YBWEM Roos, neurologist (AMC); R van den Berg, neuroradiologist (AMC); Prof CBLM Majoie, neuroradiologist (AMC); J Horn, neurointensivist (AMC); and Prof GJE Rinkel, neurologist (UMCU). New members may be added if more centers join the study.

### Data and Safety Monitoring Board

Prof BMJ Uitdehaag, clinical neuroepidemiologist (VUMC); Prof ARJ Girbes, intensivist (VUMC); and N van Geloven, statistician (AMC).

### Statistical analysis

D Verbaan, clinical epidemiologist (AMC); MR Germans, neurosurgeon (AMC); R Post, resident neurosurgery (AMC); and Prof RJ de Haan, clinical epidemiologist (AMC).

## Trial status

The study start date is 15 May 2013. The estimated study duration will be 3 years.

## Abbreviations

ALP: Alkaline phospatase; ALT: Alanine aminotransferase; AMC: Academic Medical Center; AST: Aspartate aminotransferase; CI: Confidence interval; CT: Computed tomography; CTA: Computed tomography angiography; DCI: Delayed cerebral ischemia; DSA: Digital subtraction angiography; DSMB: Data and Safety Monitoring Board; MEC: Medical Ethics Committee; MR: Magnetic resonance; mRS: Modified Rankin Scale; PI: Principal investigator; PROBE: Prospective randomized, open-label, blinded endpoint; RCT: Randomized controlled trial; SAH: Subarachnoid hemorrhage; SC: Study coordinator; TXA: Tranexamic acid; ULTRA: Ultra-early tranexamic acid after subarachnoid hemorrhage; UMCU: University Medical Center Utrecht; VUMC: VU University Medical Center; WFNS: World Federation of Neurological Surgeons; γ-GT: Gamma-glutamyltransferase.

## Competing interest

The authors declare that they have no competing interests.

## Authors’ contributions

MRG developed the conception and design of the study, and drafted the manuscript. RP and BAC critically revised the manuscript. GJER participated in the conception and design of the study, critically revised the manuscript, and is PI in one of the initiating study centers. WPV participated in the conception and design of the study, critically revised the manuscript, and is PI in two of the study centers. DV is PI, developed the conception and design of the study, and critically revised the manuscript. All authors read and approved the final manuscript.
